# Sequential monitoring of lymphocyte subsets and of T-and-B cell neogenesis indexes to identify time-varying immunologic profiles in relation to graft-versus-host disease and relapse after allogeneic stem cell transplantation

**DOI:** 10.1371/journal.pone.0175337

**Published:** 2017-04-11

**Authors:** Cristina Skert, Simone Perucca, Marco Chiarini, Viviana Giustini, Alessandra Sottini, Claudia Ghidini, Stefano Martellos, Federica Cattina, Benedetta Rambaldi, Valeria Cancelli, Michele Malagola, Alessandro Turra, Nicola Polverelli, Simona Bernardi, Luisa Imberti, Domenico Russo

**Affiliations:** 1 Chair of Haematology, Stem Cell Transplantation Unit, University of Brescia, Brescia, Italy; 2 Centro Ricerca Emato-oncologica AIL (CREA), Spedali Civili of Brescia, Brescia, Italy; 3 Department of Life Sciences, Research Unit of Biodiversity Informatics, University of Trieste, Trieste, Italy; University of Kentucky, UNITED STATES

## Abstract

T and B lymphocyte subsets have been not univocally associated to Graft-versus-host disease (GVHD) and relapse of hematological malignancies after stem cell transplantation (SCT). Their sequential assessment together with B and T cell neogenesis indexes has been not thoroughly analysed in relation to these changing and interrelated immunologic/clinic events yet.

Lymphocyte subsets in peripheral blood (PB) and B and T cell neogenesis indexes were analysed together at different time points in a prospective study of 50 patients. Principal component analysis (PCA) was used as first step of multivariate analysis to address issues related to a high number of variables versus a relatively low number of patients. Multivariate analysis was completed by Fine-Gray proportional hazard regression model. PCA identified 3 clusters of variables (PC1-3), which correlated with acute GVHD: PC1 (pre-SCT: KRECs≥6608/ml, unswitched memory B <2.4%, CD4+T_CM_ cells <45%; HR 0.5, p = 0.001); PC2 (at aGVHD onset: CD4+>44%, CD8+T_CM_ cells>4%; HR 1.9, p = 0.01), and PC3 (at aGVHD onset: CD4+TEM_RA_<1, total Treg<4, Treg_EM_ <2 cells/μl; HR 0.5, p = 0.002). Chronic GVHD was associated with one PC (Treg_EM_ <2 cells/μl at day+28, CD8+TEM_RA_<43% at day+90, immature B cells<6 cells/μl and KRECs<11710/ml at day+180; HR 0.4, P = 0.001). Two PC correlated with relapse: PC1 (pre-SCT: CD4+ <269, CD4+T_CM_ <120, total Treg <18, Treg_CM_ <8 cells/μl; HR 4.0, p = 0.02); PC2 (pre-SCT mature CD19+ >69%, switched memory CD19+ = 0 cells and KRECs<6614/ml at +90; HR 0.1, p = 0.008). All these immunologic parameters were independent indicators of chronic GVHD and relapse, also considering the possible effect of previous steroid-therapy for acute GVHD. Specific time-varying immunologic profiles were associated to GVHD and relapse. Pre-SCT host immune-microenvironment and changes of B cell homeostasis could influence GVH- and Graft-versus-Tumor reactions. The paradoxical increase of EM Treg in PB of patients with GVHD could be explained by their compartmentalization outside lymphoid tissues, which are of critical relevance for regulation of GVH reactions.

## Introduction

Long term efficacy of allogeneic stem cell transplantation (SCT) in haematological malignancies relies primarily on graft-versus-tumor (GVT), which partly overlaps with graft-versus-host disease (GVHD)[1,2], the most common cause of morbidity and mortality in SCT [3]. However, GVT and GVHD are probably characterized by different intensity of immune reactions, which can be modulated by different subsets of donor T and B lymphocytes [1–4]. Several studies correlated T lymphocyte subtypes in peripheral blood (PB) with GVHD (acute and chronic) and relapse, although without univocal results [5–18]. The role of B lymphocytes in chronic GVHD (cGVHD) was evidenced by several authors, whereas their relationship with acute GVHD (aGVHD) and relapse has been poorly investigated [5,19–26]. Adequate thymic function measured by quantification of T-cell receptor excision circles (TRECs) has been correlated with balanced immune reconstitution and reduced risk of infections [27–29]. Levels of k-deleting recombination excision circles (KRECs) have been associated with poor B lymphocyte reconstitution and cGVHD, whereas a straightforward relationship between KRECs values and aGVHD has not been evidenced yet [30–32]. The uncertain and controversial findings reported in literature could be partly explained by the difficulty of analysing all these immunologic variables in a high number of patients with an extensive monitoring in the time. Furthermore, most studies focused on one outcome only, GVHD or relapse, without considering their complex interplay. The rationale of our study relies on the following points:

1. T lymphocytes are the principal effectors and coordinators of immune responses, and B lymphocytes have an emerging role not only as effectors but also as long-lasting regulators of immune reactions [1,2,19–21]. Hence, the importance of B and T cell neogenesis indexes as well.
2. A sequential monitoring of lymphocyte subsets and thymic and bone marrow output indexes could better fit with the changing behavior of GVHD and relapse, allowing the identification of specific immunologic indicators, which could differ depending on the time before and after SCT. In particular, the start of monitoring already before SCT could allow to identify a correlation between pre-SCT host immune profiles and GVHD or relapse. In fact, the state of host immune microenvironment at SCT, which influences alloreactions by donor lymphocytes [3,19,21], may partially depend on its state pre-SCT.
3. To our knowledge, no previous study analysed all the above-mentioned variables together before and after SCT in relation to aGVHD, cGVHD and relapse.

We prospectively evaluated T and B lymphocyte subsets together with thymic and bone marrow output indexes in 50 patients at different time points before and after SCT in relation to aGVHD, cGVHD, and relapse, as clinical indicator of ineffective GVT. We used a 2-step multivariate analysis, which included principal component analysis (PCA), to counterbalance the limits of the relatively low number of enrolled patients in comparison to the high number of variables considered in this study.

## Patients and methods

### Patients and transplant procedures

Prospective evaluations of lymphocyte subsets in PB and thymic and bone marrow output indexes were performed in 50 not consecutive patients who underwent allogeneic SCT (Table 1). Acute leukaemia was the prevailing diagnosis (56%); 17 patients had lymphomas; one patient only was treated with rituximab 2 years before transplantation.

**Table 1:** Characteristics of the 50 patients and transplant.

*Characteristics*			%
*Age at SCT (years)*			
	median (range)	49 (17–66)	
*Sex*			
	Male	31	62
	Female	19	38
*Diagnosis*			
	AL	28	50
	MDS	5	10
	Lymphomas^	17	34
*Status at SCT*			
	CR/upfront	30	60
	PR*	12	24
	NR*	8	16
*Donor*			
	MUD	31	62
	MRD	19	38
*MUD-HLA match (8/8 alleles)*		30	60
*Donor sex*			
	Male	32	64
	Female	18	36
*Sex mismatch*		24	48
*Conditioning*^^			
	MAC	20	40
	RIC	30	60
*ATG*			
	Yes	29	58
	No	21	42
*Source of stem cells*			
	PB	42	84
	BM	8	16
*CD34+ cell dose (x10^6/kg)*			
	median (range)	5 (1.1–6.4)	
*CD3+ cell dose (x10^7/kg)*			
	median (range)	16.4 (1.2–41)	
*GVHD prophylaxis*			
	CyA+MTX	50	100
*Follow-up (months)*			
	median (range)	25 (3–48)	

SCT = stem cell transplantation; AL = acute leukaemia; MDS = myelodisplastic syndrome; CR = complete remission; upfront = never treated (all MDS); PR = partial remission; NR = no response; MUD = matched unrelated donor; MRD = matched related donor; MAC = myeloablative conditioning; RIC = reduced intensity conditioning; ATG = anti-thymocyte globulin; PB = peripheral blood; BM = bone marrow; CyA = cyclosporine A; MTX = methotrexate

^One patient was treated with rituximab 2 years before transplantation

*Patients in PR or NR at SCT were in complete remission at the first evaluation after SCT (day+30 for AL and MDS; day +60 for lymphomas)

^^MAC subtypes: Total body irradiation (12 Gy/6F)+cyclophosphamide (CY) (3 patients); busulphan+CY (17 patients); ^^RIC subtypes: thiotepa+CY (13 patients); thiotepa+fludarabine+CY (13 patients); busulphan+fludarabine (4 patients)

Cytomegalovirus (CMV) and HHV-6 viral replication were monitored weekly by quantitative real-time polymerase chain reaction (PCR) in plasma. Fungal infections were evaluated according to the revised criteria of EORTC/MSG Consensus Group [33]. Blood stream infection was defined according to Poutsiaka D et al. [34]. Chimerism was assessed by tandem repeats (VNTR)-PCR test on PB mononuclear cells (PBMCs), polymorphonuclear leukocytes and lymphocytes at day+30, +90, +180, and in case of disease relapse. The first evaluation of disease was performed at day +30 for acute leukaemia and myelodisplastic syndrome, and at day +60 for lymphomas. Diagnosis and grading of aGVHD and cGVHD were primarily based on clinical findings [35–37]. Whenever possible, clinical data were supported by histopathologic findings of target organs. cGVHD was defined as mild, moderate or severe according to NIH criteria for cGVHD grading [37]. GVHD prophylaxis included cyclosporine A (CyA) i.v., and a short course of methotrexate (MTX). ATG-Fresenius was used in 26 of patients with unrelated donor and 3 patients with related donor. CyA was given orally in two doses as soon as patients were able to have an oral intake. CyA was the only GVHD prophylaxis in all patients until its tapering, or until the beginning of the therapy against aGVHD or moderate/severe cGVHD. First-line therapy for acute GVHD of at least grade II included CyA and 2 mg/kg/day methylprednisone. Methylprednisone 1 mg/kg/day was the first-line therapy for cGVHD. The study was performed on a subgroup of transplanted patients (from 31/03/2011 to 01/06/2015), who were enrolled in the study “COORTE HSCT” (n.854 26/05/2010). This study was reviewed and approved by the Ethics Committee of the Hospital “Spedali Civili” of Brescia. Patients provided written consent in accordance with the Declaration of Helsinki. One minor only was included and written consent was obtained from the parents on its behalf.

From the analysis of this substudy, patients with the following characteristics were excluded or removed:

- bone marrow and PB involvement by lymphoproliferative disease at SCT;
- follow-up < 3 months;
- absence of full chimerism at day +30 or loss of chimerism independently from relapse;
- persistent disease at the first evaluation after SCT;
- steroid-refractory aGVHD development.

### Assessment of lymphocyte subsets and index of T and B cell neogenesis

To investigate a possible correlation of lymphocyte subsets and index of T and B cell neogenesis with aGVHD, cGVHD, and relapse, we performed:

1. a flow cytometry analysis of T and B cell subsets;
2. a quantification of TRECs and KRECs by Real-Time PCR.

The assessment was planned pre-SCT, at day +28, +90, +180 and at the time of aGVHD, which developed at a median time of 27 days (22–100). Hence, aGVHD analysis included only the assessment pre-SCT, at onset of aGVHD, and at day+28 for patients without it. Since cGVHD developed between the 6^th^ and the 10^th^ month (median time: 7 months), the immunologic parameters at each time point were analysed in relation to cGVHD. Relapse occurred between the 3^rd^ and the 12^th^ month (median time: 6 months), therefore the assessment of day +180 was excluded from relapse analysis.

The assessment was also performed in 15 age-matched healthy controls.

### Analysis of lymphocyte subsets by flow cytometry

#### B cell subsets

One million PBMCs were phenotyped after staining with peridin-clorophyll protein-Cy5.5 anti-CD19, phycoerythrin Cy7 anti-CD10, fluorescein isothiocyanate anti-IgD, and phycoerythrin anti-CD27 (BD Pharmingen, San Diego, CA, USA) monoclonal antibodies (mAbs). Cells were initially gated for CD19 expression, and then for CD10 marker to identify immature CD19+CD10+ B cells and mature CD19+CD10- B cells. Mature B cells were examined for IgD and CD27 molecule expression to recognize naïve IgD+CD27- B cells, unswitched memory IgD+CD27+ B cells, switched memory B cells (IgD-CD27+), and double negative B cells (DN; IgD-CD27-).

#### T cell subsets

PBMCs were stained with Horizon V500 anti-CD3, allophycocyanin-H7 anti-CD4, Horizon V450 anti-CD8, fluorescein isothiocyanate anti-CD45RA (BD Pharmingen, San Diego, CA, USA), phycoerythrin anti-CD25, peridinin-chlorophyll protein-Cy5.5 anti-CCR7 (BioLegend, San Diego, CA, USA), phycoerythrin-Cy7 anti-CD127 (eBioscience, San Diego, CA, USA), and allophycocyanin anti-CD31 (Miltenyi Biotec, Bergisch Gladbach, Germany) mAbs. PBMCs were first gated for CD3 expression, then for CD4 and CD8 markers, and finally for the expression of CD45RA and CCR7 to identify naïve CD4+/CD8+(CD45RA+CCR7+), central memory CD4+/CD8+ (T_CM_; CD45RA-CCR7+), effector memory CD4+/CD8+ (T_EM_; CD45RA-CCR7-), and terminally differentiated effector memory (TEM_RA_;CD45RA+CCR7-) cells. T regulatory cells (Treg) were identified as CD4+CD25int/highCD127low/- lymphocytes. Treg were further phenotyped as CD4+CD25int/highCD127low/-CD45RA+CCR7+ naïve Treg, CD4+CD25int/highCD127low/-CD45RA-CCR7+ Treg_CM_ and CD4+CD25int/highCD127low/-CD45RA-CCR7-Treg_EM_ subsets. Recent thymic emigrants (RTE) were recognized as naïve CD4+ lymphocytes expressing the CD31 molecule.

Absolute count and percentage were calculated for each T and B cell subset. Data were acquired using an eight-colour FACSCanto II cytometer and analysed with FACS Diva software (BD Biosciences, San Jose, CA, USA).

#### Analysis of T and B cell neogenesis by means of TRECs and KRECs quantification

Thymic and bone marrow outputs were measured from DNA from PBMCs at different time points. KRECs and TRECs were quantified simultaneously by duplex quantitative Real-Time PCR (7500 Fast Real-Time PCR of Applied Biosystems, Foster City, CA), as described elsewhere [38,39]. Their quantities were obtained from a standard curve obtained by serial dilutions of a linearized plasmid DNA, containing three inserts corresponding to fragments of KRECs, TRECs and the reference gene, which is a fragment of T-cell receptor constant alpha gene. Data are expressed as number of copies per ml of blood, equal to (KRECs or TRECs/PBMC) × (lymphocyte+monocyte count in 1 ml of blood).

#### Assessment of steroid treatment effect

In aGVHD analysis, the possible effect of steroid treatment on values of lymphocytes, TRECs, and KRECs was excluded, as they were assessed at GVHD onset before the beginning of the therapy. All patients with cGVHD were off steroid therapy at its onset. However, the influence of a previous steroid treatment for aGVHD before the occurrence of cGVHD or relapse was considered. Parameters of steroid effect were: 1. administration of steroid therapy at each time point; 2. cumulative dose of steroids (mg/kg) at day +28, from day +28 to +90, and from day +90 to +180; 3. number of days out steroids at each time point. A correlation between the parameters of steroid effect and values of lymphocyte, TRECs, and KRECs was analysed at each time point.

### Statistical analysis

Univariate analysis of variables in relation to aGVHD, cGVHD and relapse was performed by Mann-Whitney U test to compare continuous values, and chi-squared test to compare differences in percentage. Count and percentage of lymphocyte subsets, TRECs and KRECs values at the different time points were analyzed together with clinical variables (see Table 1). The analysis included infectious events before the onset of aGVHD or cGVHD and before the median time of their onset for patients without these complications. aGVHD and cGVHD were also considered for relapse analysis. The comparison of lymphocyte, TRECs, and KRECs values between patients on and off steroid therapy at each time point was also performed by Mann-Whitney test. The Spearman rank correlation analysis was used to estimate the relationship between cumulative dose of steroids or days off steroid therapy and values of the immunologic variables.

The immunologic parameters found to be significant in univariate analysis at each time point were included in the first step of multivariate analysis, which consisted in PCA, in order to solve the problem of a high number of variables in comparison with a relatively limited and heterogeneous pool of patients. PCA reduces the dimensionality of a large number of interrelated variables, while retaining as much information as possible [40]. Since PCA transforms possibly correlated variables into a smaller number of uncorrelated variables (principal components, PCs), each PC is a cluster of correlated variables. The first PC (PC1) accounts for the largest part of the total variance in the dataset; the second (PC2) accounts for the second greatest amount of the variance, and so on. The last few PCs do not account for much of the variance, and therefore can be ignored. The eigenvalue-one criterion (Kaiser criterion) was used for extracting relevant PCs (eigenvalue >1). Variables with component loading > 0.5 (absolute value) only were included in each PC. Loadings vary in value from -1 to 1 and represent the degree to which each of the variables correlates with each PC.

Multivariate analysis was completed by Fine-Gray proportional hazard regression model for competing events [41,42], which included:

1. PCs scores (values of the PCs extracted by PCA for each patient);
2. clinical variables and parameters of steroid effect, which were significant in univariate analysis.

For the immunologic variables clustered in each PC, which resulted to be significant in this second step of multivariate analysis, the median value was calculated and taken as the cut point.

Death without GVHD and death without relapse were the competing events for GVHD and relapse analysis, respectively. All p values were 2-sided and p<0.05 was considered statistically significant.

## Results

### Values of lymphocyte subsets, TRECs, and KRECs in relation to patient and transplant characteristics

All immunological variables did not differ depending on age, sex or disease status. Patients with diagnosis of lymphoma showed lower pre-SCT values (median, range) of naïve CD4+ (14, 1–90 vs 56, 9–205; p = 0.003), RTE (9, 0–72 vs 30, 5–94; p = 0.02), naïve Treg (1, 0–7 vs 3, 0–12; p = 0.03), CD4+TEM_RA_ cells (40, 0–110 vs 78, 1–469; p = 0.02), and immature B cells (5, 0–32 vs 15, 0–95; p = 0.03) in comparison to other diagnosis. Among transplant variables, the use of ATG only was associated with lower values of naïve CD4+ and RTE from day+28 to day +180; naïve Treg and CD4+TEM_RA_ cells remained lower at day +28 and +90 (S1 Table).

### Clinical and immunological characteristics of patients with aGVHD

28 patients (56%) developed aGVHD (median time: 27 days; range, 22–100); it was of at least grade II in 20 patients (grade III in 2 out of 20 patients). Before its onset, 6 patients (21%) had bacterial infections. CMV reactivations, without end-organ disease, and fungal infections were observed in 7 (25%) and 4 patients (14%), respectively. Patients with and without aGVHD did not differ in clinical and transplant characteristics (S2 Table). No significant differences were observed as far as immunosuppressive prophylaxis (use of ATG, MTX doses, CyA blood levels) and type of reduced intensity or myeloablative conditioning were concerned (data not shown). Univariate analysis of immunological variables is summarized in Table 2.

**Table 2:** Comparison of immunological variables between patients with and without acute GVHD.

Immunological variables ^	Pre-SCT			aGVHD onset*		
	a GVHD			a GVHD		
	Yes	No	p	Yes	No	p
CD4+(total)	268 (57–1215)	228 (4–1118)	0,59	100 (1–1174)	24 (0–405)	0,07
RTE	10 (3–123)	25 (0–94)	0,64	4 (0–115)	0 (0–24)	**0,01**
CD4+naïve	37 (4–316)	56 (1–205)	0,84	8 (0–165)	0 (0–34)	**0,005**
CD4+T_CM_	113 (37–1092)	98 (2–233)	0,30	25 (0–254)	8 (0–86)	**0,03**
CD4+T_EM_	63 (2–210)	59 (2–769)	0,90	45 (0–986)	16 (0–318)	0,26
CD4+TEM_RA_	2 (0–14)	2 (0–209)	0,66	2 (0–36)	0 (0–20)	**0,02**
Treg (total)	16 (3–429	17 (1–45)	0,97	7 (0–30)	2 (0–11)	**0,005**
Treg naïve	1 (0–19)	3 (0–12)	0,41	2 (0–6)	0 (0–5)	**0,02**
Treg_CM_	8 (2–18)	5 (0–21)	0,29	3 (0–17)	0 (0–4)	**0,006**
Treg_EM_	6 (0–25)	6 (1–22)	0,90	3 (0–17)	1 (0–7)	**0,01**
CD8+(total)	151 (10–870)	147 (1–998)	0,84	44 (1–1109)	30 (0–1138)	0,27
CD8+ naïve	17 (1–126)	16 (0–105)	0,80	4 (0–85)	2 (0–21)	0,25
CD8+T_CM_	11 (1–60)	9 (0–83)	0,95	3 (0–34)	1 (0–17)	0,08
CD8+T_EM_	63 (1–520)	29 (0–566)	0,30	26 (0–360)	21 (0–669)	0,50
CD8+TEM_RA_	44 (1–446)	65 (0–469)	0,82	8 (0–603)	5 (0–434)	0,37
CD19+(total)	22 (0–102)	32 (0–145)	0,50	1 (0–11)	0 (0–19)	0,27
Immature CD19+	4 (0–38)	9 (0–95)	0,19	0 (0–7)	0 (0–6)	0,81
Mature CD19+	10 (0–93)	16 (0–100)	0,88	1 (0–11)	0 (0–15)	0,40
CD19+ naïve	5 (0–50)	4 (0–93)	0,98	0 (0–9)	0 (0–11)	0,70
UM CD19+	0 (0–12)	0 (0–4)	0,41	0 (0–1)	0	0,40
SM CD19+	1 (0–24)	1 (0–7)	0,67	0 (0–2)	0 (0–1)	0,80
M/DN CD19+	0 (0–7)	1 (0–5)	0,50	0 (0–1)	0 (0–1)	0,85
TRECs/ml	63 (0–1360)	57 (0–1960)	0,78	82 (0–1769)	23 (0–885)	0,34
KRECs/ml	5099 (0–23957)	9319 (0–79533)	**0,04**	191 (0–3914)	78 (0–9764)	0,59

SCT = stem cell transplantation; aGVHD = acute GVHD; RTE = recent thymic emigrants; CM = central memory; EM = effector memory; TEMRA = terminally differentiated effector memory; UM = unswitched memory; SM = switched memory; M/DN = memory double negative; TRECs = T-cell receptor excision circles; KRECs = k-deleting recombination excision circles

^ Lymphocyte counts are expressed as cells/μl (median; range). Significant differences in percentage of lymphocyte subsets are reported in the section “Results”.

*Values at day+28 were analysed for patients without aGVHD.

Significant differences in percentages of lymphocyte subsets are reported in the text below. Patients with aGVHD had: a) before SCT, higher percentages of CD4+T_CM_ (49 vs 41; p = 0.02) and unswitched memory B cells (4 vs 2; p = 0.04) and lower values of KRECs (p = 0.04); b) at GVHD onset, higher values of RTE, naïve CD4+, CD4+T_CM_, CD4+TEM_RA_ cells, and all Treg subtypes (p<0.05), and an increased percentage of CD4+ (49 vs 28; p = 0.006) and CD8+T_CM_ cells (7 vs 4; p = 0.04).

### Multivariate analysis of variables associated with aGVHD

In the 2-step multivariate analysis, three PC were evidenced as independent factors associated with aGVHD, also considering the possible effect of lymphoma diagnosis and use of ATG (Table 3). PC1 was inversely correlated with aGVHD (HR 0.5; p = 0.001), and included pre-SCT values of KRECs, percentages of unswitched memory B (<2.4%) and CD4+T_CM_ cells (<45%). PC2 was associated with aGVHD onset (HR 1.9; p = 0.01), and included percentages of CD4+ and CD8+T_CM_ cells. PC3 was inversely correlated with aGVHD (HR 0.5; p = 0.002), and included lower values of CD4+TEM_RA_ cells, total Treg and Treg_EM_.

**Table 3:** Clusters of immunologic variables correlated to aGVHD, cGVHD, and relapse by multivariate analysis.

	Pre-SCT		Day +28 or at aGVHD onset^		Day+90		Day+180
**aGVHD**	***PC1***	***CD4+T***_***CM***_ ***<45%***	***PC2***	***CD4+>44%***			
		***CD19+UM<2*.*4%***		***CD8+T***_***CM***_***>4%***			
		***KRECs/ml ≥6608)***					
	*HR*	*0*.*5 (0*.*3–0*.*7)*	*HR*	*1*.*9 (1*.*1–3*.*3)*			
		*p = 0*.*001*		*p = 0*.*01*			
			***PC3***	***Treg<4***			
				***Treg***_***EM***_***<2***			
				***CD4+TEM***_***RA***_***<1***			
			*HR*	*0*.*5 (0*.*3–0*.*8)*			
				*p = 0*.*002*			
**cGVHD**			***PC1***	***Treg***_***EM***_***<2***	***CD8+TEM***_***RA***_***<43%***		***ImmatureCD19+<6***
			*HR*	*0*.*4 (0*.*2–0*.*7)*			***KRECs/ml<11710)***
				*p = 0*.*001*			
**Relapse**	***PC1***	***CD4+<269***					
		***CD4+T***_***CM***_ ***<120***					
		***Treg <18***					
		***Treg***_***CM***_ ***<8***					
	*HR*	*4*.*0 (1–15*.*2)*					
		*p = 0*.*02*					
	***PC2***	***Mature CD19+ >69%***			***PC2***	***CD19+SM = 0***	
						***KRECs/ml <6614***	
	*HR*	*0*.*1 (0*.*03–0*.*6)*			*HR*	*0*.*1 (0*.*03–0*.*6)*	
		*p = 0*.*008*				*p = 0*.*008*	

SCT = stem cell transplantation; aGVHD = acute GVHD; PC = principal component; KRECs = k-deleting recombination excision circles; cGVHD = chronic GVHD; UM = unswitched memory; CM = central memory; HR = hazard ratio; EM = effector memory; TEMRA = terminally differentiated effector memory

^in aGVHD analysis, day +28 was considered as time point for patients without aGVHD; in cGVHD and relapse analysis, day+28 was the time point for all patients.

Three clusters of variables (PC1, PC2, PC3) were associated to aGVHD; one PC correlated to cGVHD; 2 PC (PC1, PC2) correlated to relapse. Lymphocyte subsets and index of B cell neogenesis included in each PC are enclosed within brackets. For the immunologic variables clustered in each PC, the median value was calculated and taken as the cut point.

### Values of lymphocyte subsets, TRECs, and KRECs in patients on steroid therapy

Patients on steroid therapy at day +28 had higher Treg_CM_ median values (cells/μl: 5, range 0–11 vs 1, range 0–17; p = 0,02) in comparison with patients out steroids, with a weak correlation with their cumulative dose (r = 0,44; p = 0,009). At day +90, decreased values of CD4+T_CM_, CD4+T_EM_, CD4+TEM_RA_, Treg_EM_, all CD8+T subtypes, all CD19+ cells, except for memory and DN B cells, and KRECs were observed in patients on steroid therapy at day+28 (Table 4). The cumulative dose of steroids at day +90 was associated to decreased values of CD4+T_CM_, CD4+T_EM_, Treg_EM_, all CD8+T and CD19+ subtypes, except for CD8+TEM_RA_ and memory unswitched B cells, although with a weak correlation (Table 4). TRECs/ml at day +180 were higher in patients without steroid treatment in the first 6 months after SCT (48, range 0–2048 vs 3, range 0–41; p = 0,01).

**Table 4:** Steroid effect on lymphocyte subsets and index of B/T cell neogenesis at day +90.

Immunologic variables^	On steroid at day +28			Steroid (C.D.)		On steroid at day +90			Steroid (C.D.)	
				day +28					day +90	
	Yes	No	p	(r*)	p	Yes	No	p	(r*)	P
RTE	1 (0–6)	1 (0–90)	0.84			2 (0–9)	1 (0–90)	0.50		
CD4+naïve	3 (0–8)	1 (0–129)	0.66			3 (0–20)	1 (0–128)	0.97		
CD4+T_CM_	10 (1–39)	35 (0–182)	**0.03**	-0.39	**0.02**	13 (1–39)	37 (0–182)	**0.02**	-0.44	**0.01**
CD4+T_EM_	6 (3–30)	46 (0–415)	**0.005**	-0.50	**0.003**	23 (3–52)	50 (0–415)	**0.01**	-0.45	**0.009**
CD4+TEM_RA_	1 (0–2)	2 (0–49)	**0.01**	-0.44	**0.01**	1 (0–26)	2 (0–49)	0.17		
Treg naïve	0 (0)	0 (0–7)	0.13			0 (0–19	0 (0–7)	0.55		
Treg_CM_	1 (0–45)	2 (0–13)	0.39			1 (0–45)	2 (0–13)	0.53		
Treg_EM_	1 (0–4)	4 (0–11)	**0.02**	-0.43	**0.01**	2 (0–4)	4 (0–119	**0.006**	-0.47	**0.007**
CD8+ naïve	2 (0–6)	8 (0–73)	**0.01**	-0.40	**0.02**	2 (0–11)	10 (0–73)	**0.02**	-0.40	**0.02**
CD8+T_CM_	0 (0–7)	7 (0–85)	**0.01**	-0.44	**0.01**	2 (0–7)	8 (0–85)	**0.007**	-0.52	**0.002**
CD8+T_EM_	13 (1–51)	54 (0–1264)	**0.04**	-0.37	**0.04**	18 (1–57)	62 (0–1264)	**0.04**	-0.39	**0.03**
CD8+TEM_RA_	8 (0–44)	45 (0–947)	**0.02**	-0.39	**0.03**	21 (0–71)	52 (0–947)	0.05		
Immature CD19+	0 (0)	6 (0–122)	**0.03**	-0.39	**0.02**	0 (0–8)	7 (0–122)	**0.006**	-0.51	**0.003**
Mature CD19+	0 (0–3)	11 (0–231)	**0.008**	-0.47	**0.006**	1 (0–23)	14 (0–231)	**0.01**	-0.47	**0.007**
CD19+ naïve	0 (0–2)	8 (0–210)	**0.03**	-0.38	**0.03**	0 (0–19)	8 (0–210)	**0.01**	-0.48	**0.005**
UM CD19+	0 (0)	0 (0–1)	0.57			0 (0)	0 (0–2)	0.48		
SM CD19+	0 (0)	1 (0–13)	*0*.*06*			0 (0–2)	1 (0–13)	**0.02**	-0.44	**0.01**
M/DN CD19+	0 (0)	1 (0–15)	*0*.*08*			0 (0–1)	1 (0–15)	**0.02**	-0.43	**0.01**
TRECs/ml	19 (4–76)	10 (0–899)	0.22			28 (0–39)	10 (0–899)	0.73		
KRECs/ml	36 (14–63)	8390 (8–96178)	**0.01**	-0.44	**0.01**	51 (11–13869)	6614 (8–96178)	0.53		

^ Lymphocyte counts are expressed as cells/μl (median; range).

*r = Spearman rank correlation coefficient (significant values only are reported)

C.D. = cumulative dose; RTE = recent thymic emigrants; CM = central memory; EM = effector memory; TEMRA = terminally differentiated effector memory; UM = unswitched memory; SM = switched memory; M/DN = memory double negative; TRECs = T-cell receptor excision circles; KRECs = k-deleting recombination excision circles

Parameters of steroid effects at each time point (day+28, +90, +180) were: 1.administration of steroid therapy; 2. cumulative dose of steroids (mg/kg); 3. number of days out steroids. These parameters were correlated to values of immunologic variables at each time point. Steroid effect parameters, which correlated to values of immunologic variables at day +28 and +180, are reported in the section “Results”.

### Clinical and immunological characteristics of patients with cGVHD

The cumulative incidence of cGVHD was 26,5% (95% C.I. 15–45). It was mild in 1, moderate in 8 and severe in 4 patients according to NIH criteria for cGVHD grading.aGVHD preceded cGVHD in 11 patients (grade ≥II in 4 patients). Before cGVHD onset, patients had 40% bacterial infections and 20% CMV reactivation without end-organ disease. In univariate analysis, matched related donor (62% vs 28%; p = 0,03) and previous aGVHD (85% vs 44%; p = 0,01) were the only clinical variables associated to cGVHD (S3 Table). Values of immunological variables in patients with and without cGVHD are shown in Table 5. As for aGVHD analysis, differences in percentages are reported in the text.

**Table 5:** Comparison of immunological variables between patients with and without chronic GVHD.

Immunological variables ^	Pre-SCT			Day+28			Day+90			Day+180		
	cGVHD			cGVHD			cGVHD			cGVHD		
	Yes	No	p	Yes	No	p	Yes	No	p	Yes	No	p
CD4+(total)	314 (146–639)	228 (4–1215)	0,51	73 (28–1174)	25 (0–503)	0,07	50 (15–663)	82 (0–382)	0,78	253 (56–552)	142 (4–506)	0,06
RTE	39 (8–123)	24 (0–94)	0,51	5 (0–26)	0 (0–115)	**0,04**	3 (0–46)	0 (0–90)	0,25	9 (0–65)	8 (0–48)	0,62
CD4+ naive	87 (11–316)	42 (1–205)	0,30	9 (1–38)	1 (0–164)	**0,02**	7 (0–72)	1 (0–129)	0,24	12 (1–69)	12 (0–65)	0,52
CD4+T_CM_	130 (94–254)	99 (2–1092)	0,11	30 (13–114)	8 (0–254)	0,05	17 (4–148)	34 (0–182)	0,63	121 (22–178)	60 (2–159)	0,07
CD4+T_EM_	59 (2–94)	59 (2–769)	0,47	26 (11–986)	16 (0–132)	0,25	37 (4–415)	43 (0–203)	0,86	162 (16–304)	60 (2–247)	0,06
CD4+TEM_RA_	2 (0–9)	5 (80–209)	0,22	2 (0–35)	0 (0–20)	0,14	3 (0–28)	2 (0–49)	0,58	15 (1–48)	3 (0–99)	0,18
Treg (total)	22 (6–42)	16 (1–45)	0,18	8 (2–17)	2 (0–27)	**0,02**	4 (0–26)	6 (0–49)	0,70	15 (2–50)	11 (0–34)	0,19
Treg naive	4 (0–19)	3 (0–12)	0,56	0 (0–2)	0 (0–6)	0,95	0 (0–3)	0 (0–7)	1,0	0 (0–3)	1 (0–6)	0,77
Treg_CM_	9 (2–13)	5 (0–21)	0,11	3 (0–9)	1 (0–17)	0,06	1 (0–13)	1 (0–45)	0,77	5 (0–17)	3 (0–11)	0,05
Treg_EM_	7 (0–24)	5 (0–22)	0,44	4 (1–14)	1 (0–8)	**0,009**	3 (0–11)	4 (0–11)	0,82	4 (0–36)	6 (0–24)	0,94
CD8+(total)	190 (109–998)	139 (1–870)	0,23	25 (5–1138)	36 (0–398)	0,43	82 (3–1918)	99 (0–2083)	0,69	369 (48–2564)	244 (0–1153)	0,34
CD8+ naive	27 (18–126)	15 (0–105)	0,06	5 (1–37)	2 (0–21)	0,15	4 (1–73)	6 (0–67)	0,95	35 (3–170)	14 (0–117)	0,37
CD8+T_CM_	14 (3–60)	9 (0–83)	0,38	2 (1–21)	1 (0–11)	0,12	2 (0–62)	7 (0–85)	0,27	20 (3–104)	18 (0–73)	0,54
CD8+T_EM_	63 (10–566)	50 (0–520)	0,51	17 (1–669)	21 (0–260)	0,63	18 (1–884)	51 (0–1264)	0,50	121 (26–985)	73 (0–438)	0,44
CD8+TEM_RA_	78 (25–370)	40 (0–469)	0,33	5 (2–803)	6 (0–122)	0,56	46 (0–947)	26 (0–712)	0,57	112 (15–1694)	82 (0–561)	0,29
CD19+(total)	36 (3–66)	31 (0–145)	0,89	1 (0–19)	1 (0–8)	0,33	1 (0–350)	16 (0–149)	0,78	60 (0–273)	25 (0–336)	0,12
Immature CD19+	15 (0–32)	8 (0–95)	0,94	0 (0–6)	0 (0–7)	0,67	0 (0–122)	6 (0–60)	0,40	26 (0–71)	3 (0–92)	**0,02**
Mature CD19+	12 (2–58)	16 (0–100)	0,89	1 (0–13)	0 (0–5)	0,27	1 (0–231)	10 (0–90)	0,65	51 (0–205)	21 (0–251)	0,23
CD19+ naïve	4 (1–30)	5 (0–92)	0,91	0 (0–11)	0 (0–1)	0,54	0 (0–210)	7 (0–74)	0,35	44 (0–191)	16 (0–225)	0,17
UM CD19+	1 (0–9)	0 (0–12)	0,24	0	0 (0–1)	0,54	0 (0–2)	0 (0–2)	0,85	1 (0–4)	1 (0–9)	0,96
SM CD19+	1 (0–23)	1 (0–24)	0,61	0 (0–1)	0 (0–2)	0,76	0 (0–8)	1 (0–13)	0,29	3 (0–19)	2 (0–22)	0,72
M/DN CD19+	0 (0–7)	1 (0–6)	0,19	0 (0–1)	0 (0–1)	0,80	0 (0–15)	1 (0–5)	0,65	2 (0–9)	1 (0–7)	0,96
TRECs/ml	101 (0–614)	31 (0–1960)	0,89	127 (10–411)	22 (0–1769)	**0,01**	20 (0–259)	7 (0–899)	0,21	3 (0–917)	38 (0–2048)	0,17
KRECs/ml	9161(233–28919)	6608(0–79533)	0,77	230 (0–9764)	89 (0–2319)	0,49	297 (11–96178)	7666 (8–58980)	0,75	28274(9–295083)	9647(14–99309)	**0,04**

^ Lymphocyte counts are expressed as cells/μl (median; range). Significant differences in percentage of lymphocyte subsets are reported in the section “Results”. RTE = recent thymic emigrants; CM = central memory; EM = effector memory; TEMRA = terminally differentiated effector memory; UM = unswitched memory; SM = switched memory; M/DN = memory double negative; TRECs = T-cell receptor excision circles; KRECs = k-deleting recombination excision circles

Patients developing cGVHD had:

1. before SCT, lower percentage of CD4+T_EM_ (15 vs 35; p = 0.04) and CD4+TEM_RA_ cells (0 vs 2; p = 0.003);
2. at day +28, higher values of RTE, naïve CD4+ cells, total Treg, Treg_EM_, and TRECs (p<0.05);
3. at day +90, different percentages of CD4+T_CM_ (24 vs 38; p = 0.02), CD8+T_EM_ (23 vs 47; p = 0.01), and CD8+TEM_RA_ cells (52 vs 35; p = 0.03);
4. at day +180, higher values of immature B cells (p = 0.02) and KRECs (p = 0.04).

### Multivariate analysis of variables associated to cGVHD

aGVHD was confirmed as clinical variable associated to cGVHD (HR 2.4 95% C.I. 1.3–41; p = 0.002), while diagnosis of lymphoma, use of ATG, and parameters of steroid effect did not show a significant influence. Only one PC correlated with the onset of cGVHD (HR 0.4 95% C.I. 0.2–0.7; p = 0.002) and it clustered the following variables: Treg_EM_ values at day +28, percentage of CD8+TEM_RA_ at day+90, values of immature B cells and KRECs at day+180 (Table 3).

#### Univariate and multivariate analysis of variables associated to relapse

Cumulative incidence of relapse was 23% (C.I. 13–42) and its rate prevailed in cases of acute myeloid leukaemia (73% vs 31%; p = 0,01). Other clinical characteristics did not correlate with relapse in univariate analysis (S4 Table), while the following immunologic variables characterized relapsed patients (Table 6):

1. before SCT, lower values of CD4+, CD4+T_CM_, Treg_CM_ cells, and lower percentages of mature (31 vs 71; p = 0.03) and memory (switched and unswitched) B cells (4 vs 23; 1 vs 4; p<0.05);
2. at day +28, lower percentage of CD4+ cells (31 vs 47; p = 0.03);
3. at day +90, higher values of switched memory B cells and KRECs, and lower values of TRECs (p<0.05).

**Table 6:** Univariate analysis of immunological variables associated with relapse.

Immunological variables ^	Pre-SCT			Day+28			Day+90		
	relapse			relapse			relapse		
	Yes	No	p	Yes	No	P	Yes	No	p
CD4+(total)	128 (4–290)	312 (33–1215)	**0,04**	87 (0–339)	45 (0–1174)	0,70	83 (0–343)	62 (4–663)	0,26
RTE	19 (0–57)	39 (3–123)	0,25	0 (0–24)	0 (0–115)	0,64	1 (0–49)	0 (0–90)	0,91
CD4+naïve	38 (1–98)	56 (3–316)	0,27	1 (0–34)	1 (0–165)	0,88	2 (0–64)	1 (0–129)	0,94
CD4+T_CM_	54 (2–123)	130 (20–1092)	**0,006**	14 (0–111)	17 (0–254)	0,57	37 (0–107)	21 (1–182)	0,28
CD4+T_EM_	35 (2–127)	69 (2–770)	0,19	26 (0–204)	17 (0–986)	0,93	52 (0–203)	36 (2–415)	0,26
CD4+TEM_RA_	1 (0–7)	3 (0–209)	0,12	1 (0–20)	1 (0–36)	0,72	2 (0–49)	2 (0–41)	0,65
Treg (total)	14 (1–19)	19 (3–45)	0,06	2 (0–30)	4 (0–27)	0,53	6 (0–18)	4 (0–49)	0,61
Treg naïve	3 (0–7)	2 (0–19)	1,0	0 (0–5)	0 (0–6)	0,53	0 (0–7)	0 (0–6)	0,72
Treg_CM_	3 (0–8)	8 (2–21)	**0,02**	1 (0–11)	1 (0–17)	0,88	2 (0–5)	1 (0–45)	0,66
Treg_EM_	5 (1–9)	7 (0–25)	0,13	2 (0–17)	2 (0–14)	0,51	5 (0–11)	3 (0–12)	0,25
CD8+(total)	147 (1–749)	151 (10–998)	0,57	48 (0–426)	28 (0–1138)	0,95	149 (0–1058)	92 (1–2083)	0,51
CD8+ naïve	2 (0–54)	17 (1–126)	0,15	7 (0–85)	3 (0–37)	0,97	8 (0–17)	6 (0–73)	0,78
CD8+T_CM_	6 (0–29)	12 (1–83)	0,12	3 (0–34)	2 (0–21)	0,92	7 (0–329	4 (0–85)	0,61
CD8+T_EM_	29 (0–286)	53 (1–566)	0,31	26 (0–260)	20 (0–669)	0,90	80 (0–470)	45 (1264)	0,42
CD8+TEM_RA_	35 (0–446)	49 (1–469)	0,61	12 (0–122)	6 (0–803)	0,90	39 (0–540)	27 (0–947)	0,91
CD19+(total)	31 (0–145)	26 (0–132)	0,70	0 (0–8)	1 (0–19)	0,87	31 (0–149)	3 (0–350)	0,08
Immature CD19+	20 (0–71)	4 (0–95)	0,23	0 (0–7)	0 (0–6)	0,78	8 (0–60)	0 (0–122)	0,09
Mature CD19+	6 (0–72)	14 (0–100)	0,98	0 (0–3)	1 (0–13)	0,44	23 (0–90)	3 (0–231)	0,09
CD19+ naïve	5 (0–71)	4 (0–93)	0,63	0	0 (0–11)	0,56	19 (0–74)	2 (0–210)	0,07
UM CD19+	0 (0–1)	0 (0–12)	0,77	0	0 (0–1)	0,58	0 (0–2)	0 (0–2)	0,90
SM CD19+	0 (0–7)	1 (0–24)	0,08	0 (0–1)	0 (0–2)	0,12	3 (0–13)	0 (0–9)	**0,01**
M/DN CD19+	0 (0–3)	1 (0–7)	0,42	0 (0–1)	0 (0–1)	0,84	2 (0–5)	0 (0–15)	0,14
TRECs/ml	31 (0–1478)	63 (0–1960)	0,54	30 (0–885)	41 (0–1769)	0,55	0 (0–899)	17 (0–558)	**0,03**
KRECs/ml	7859 (0–43299)	6508 (0–79533)	0,66	86 (0–2319)	107 (0–9764)	0,92	9215(42–36588)	1130(8–96178)	**0,04**

^ Lymphocyte counts are expressed as cells/μl (median; range). Significant differences in percentage of lymphocyte subsets are reported in the section “Results”. RTE = recent thymic emigrants; CM = central memory; EM = effector memory; TEMRA = terminally differentiated effector memory; UM = unswitched memory; SM = switched memory; M/DN = memory double negative; TRECs = T-cell receptor excision circles; KRECs = k-deleting recombination excision circles

In multivariate analysis, 2 PC correlated with relapse (Table 3):

- PC1 including pre-SCT CD4+, CD4+T_CM_, all Treg and Treg_CM_ cells (HR 4.0; p = 0.02);
- PC2 including pre-SCT mature CD19+, switched memory CD19+ cells (+90) and KRECs (+90) (HR 0.1; p = 0.008).

## Discussion

T and B lymphocyte subsets in PB have been investigated as potential markers of aGVHD and cGVHD or relapse without univocal results [5–18,22–26]. TRECs and KRECs values have been mainly investigated in relation to poor immune reconstitution and risk of infections as consequence of GVHD. Their role as predictors of GVHD and relapse is not well established yet [27–32]. These controversial findings could be related to analytic approaches, which did not consider the variability over time of the complex interplay among all these immunologic variables, GVHD, and GVT together. Furthermore, a sufficiently large and homogeneous pool of patients may be difficult to obtain in the transplant setting in a relatively short time, in order to perform such a comprehensive analysis. We addressed these issues performing a prospective analysis of T and B lymphocyte subsets, TRECs and KRECs values at different time points before and after SCT, in relation to aGVHD, cGVHD, and relapse. A 2-step multivariate analysis including PCA was used to solve the problem of a relatively limited and heterogeneous pool of patients in comparison to the numerous variables. PCA has important noise-reducing properties in small populations of patients, and can achieve the same noise reduction as using large populations [40]. In this 2-step multivariate analysis, specific time-varying immunologic profiles were correlated at each outcome of the study: aGVHD, cGVHD, and relapse.

aGVHD was associated with an immunologic profile including pre-transplant lower KRECs values and some lymphocyte subtypes pre-SCT and at its onset. Lower KRECs values may indicate a damaged bone marrow microenvironment, predisposing to an impairment of central tolerance, and eventually to an imbalance of B cells. This may promote the prevalence of recipient B lymphocytes with pathogenic functions such as secretion of pro-inflammatory cytokines and, as antigen presenting cells (APC), activation of effector T cells or inhibition of Treg [43–45]. These host B-APC could survive after SCT, and stimulate donor effector T cells in GVHD reactions. Before SCT, the prevalence in PB of unswitched memory over the other mature B cells could originate from an impairment of germinal center function, as other checkpoint for B tolerance, contributing to recipient B cell imbalance [43]. Both pathogenic and protective effects of recipient B lymphocytes on aGVHD have been reported, mostly from studies performed on mice models, or from studies without distinction among B cell subtypes [19,21].

At the onset of aGVHD, patients had a higher percentage of CD8+T_CM_ and increased values of CD4+TEM_RA_ cells, “senescent” cells with raised levels of activation, resistant to apoptosis [46]. Their increase, never reported by other authors in aGVHD, may be the expression of dysregulated and skewed response toward host antigens, and could partly explain the resistance to aGVHD therapy. Patients with aGVHD showed also higher PB values of Treg_EM_. This apparently contradictory finding was rarely reported before [5,7]. Several studies have been performed in murine models of GVHD, or in transplanted patients without distinction among Treg subsets, which display different regulatory efficiency [47]. Treg_EM_ seem less effective than naïve Treg, since naïve Treg may directly inhibit effector T cells and APC in lymphoid tissues, which are of critical relevance for induction and suppression of GVH reactions [48,49]. Unlike naïve Treg, Treg_EM_ lack of homing receptors such as L-selectin and chemokine receptor 7 (CCR7), which are needed for migration to secondary lymphoid organs and in situ regulation of immune responses [48–50]. On the other hand, increased Treg_EM_ in PB could be expression of an ineffective compensatory reaction.

The immunologic profile associated with cGVHD included Treg_EM_ (+28), CD8+TEM_RA_ cells (+90), and immature B cells and KRECs values (+180). Higher PB values of Treg_EM_ at day +28 were associated with cGVHD development, highlighting their potential role as early indicator of dysregulated immune responses. The prevalence of TEM_RA_ cells among CD8+ lymphocytes before cGVHD onset (day+90) may be another indicator of dysregulated and skewed immune system. Such dysregulation may involve the B-compartment as well. Indeed, we observed increased values of KRECs and immature B cells at day+180 in patients with cGVHD, as possible expression of hyper-stimulated B cell output, defective censoring of host-reactive B cells in bone marrow, and defective maturation to naïve B cells in secondary lymphoid organs. Increased values of KRECs in cGVHD were never reported before, while there are few studies on the impact of GVHD on B cell generation [30–32]. The prevalence of immature B cells in PB was evidenced in autoimmune diseases and immunodeficiences, while their association with cGVHD is controversial [22,23,25,44,45]. Immature B cells elicit rapid antibody-dependent or -independent responses in absence of T cell induction, and are not completely depleted by CD20-targeted immunotherapy [51], partly explaining the incomplete responses observed in cGVHD.

Relapse, as index of ineffective GVT, showed also its typical immunologic hallmarks, depending on the time related to SCT. Relapsed patients had an ineffective T- immune system with decreased values of CD4+, CD4+T_CM_, Treg, and Treg_CM_ cells already before transplantation. We showed a possible role of different B cell subsets in relapse control both before and after SCT, while few evidences, mostly regarding antibodies production and GVT, were reported before [5,19]. The decreased percentage of recipient mature B cells in relapsed patients suggests a defect of B-immune system even before SCT.

At day+90, ineffective GVT correlated with increased values of KRECs and switched memory B cells, as possible expression of an early but ineffective B cell-hyperactivation.

Different clusters of immunological parameters at different time points were evidenced as indicators of aGVHD, cGVHD and relapse, allowing a clear-cut distinction between these immunological/clinical events. As a novel finding, we highlighted a possible role of pre-transplant host immune-microenvironment in promoting or dampening GVHD and GVT. The atypical association of Treg_EM_ with GVHD could be explained by the different efficiency of Treg subsets. Imbalances of B-cell homeostasis appeared to be involved both in GVHD and relapse with different indicators and features, also depending on the time before or after SCT. These specific time-varying immunologic profiles could drive a targeted, time-varying modulation of both immunosuppressive prophylaxis and pre-emptive therapy. Although the 2-step multivariate analysis addresses issues related to an unfavourable ratio between number of patients and number of variables, further studies may be helpful to validate our findings.

## Supporting information

S1 Minimal Dataset (XLS)

S1 Table ATG and values of T-lymphocyte subsets at different time points after SCT. SCT = stem cell transplantation; RTE = recent thymic emigrants; TEMRA = terminally differentiated effector memory; ^ Lymphocyte counts are expressed as cells/μl (median; range).
(DOC)

S2 Table Comparison of clinical and transplant characteristics between patients with and without acute GVHD. aGVHD = acute GVHD; SCT = stem cell transplantation; AL = acute leukaemia; MDS = myelodisplastic syndrome; CR = complete remission; upfront = never treated (all MDS); PR = partial remission; NR = no response; MUD = matched unrelated donor; MRD = matched related donor; MAC = myeloablative conditioning; RIC = reduced intensity conditioning; ATG = anti-thymocyte globulin; PB = peripheral blood; BM = bone marrow. *Patients in PR or NR at SCT were in complete remission at the first evaluation after SCT (day+30 for AL and MDS; day +60 for lymphomas)
(DOC)

S3 Table Comparison of clinical and transplant characteristics between patients with and without chronic GVHD. cGVHD = chronic GVHD; SCT = stem cell transplantation; AL = acute leukaemia; MDS = myelodisplastic syndrome; CR = complete remission; upfront = never treated (all MDS); PR = partial remission; NR = no response; MUD = matched unrelated donor; MRD = matched related donor; MAC = myeloablative conditioning; RIC = reduced intensity conditioning; ATG = anti-thymocyte globulin; PB = peripheral blood; BM = bone marrow; aGVHD = acute GVHD; ^49 evaluable patients (surviving more than 3 months) *Patients in PR or NR at SCT were in complete remission at the first evaluation after SCT (day+30 for AL and MDS; day +60 for lymphomas)
(DOC)

S4 Table Comparison of clinical and transplant characteristics between relapsed and non relapsed patients. SCT = stem cell transplantation; AML = acute myeloid leukaemia; ALL = acute lymphoblastic leukaemia; MDS = myelodisplastic syndrome; CR = complete remission; upfront = never treated (all MDS); PR = partial remission; NR = no response; MUD = matched unrelated donor; MRD = matched related donor; MAC = myeloablative conditioning; RIC = reduced intensity conditioning; ATG = anti-thymocyte globulin; PB = peripheral blood; BM = bone marrow; aGVHD = acute GVHD; cGVHD = chronic GVHD. *Patients in PR or NR at SCT were in complete remission at the first evaluation after SCT (day+30 for AL and MDS; day +60 for lymphomas)
(DOC)
