# Supplementary material for: Sequential monitoring of lymphocyte subsets and of T-and-B cell neogenesis indexes to identify time-varying immunologic profiles in relation to graft-versus-host disease and relapse after allogeneic stem cell transplantation
Source: PLoS One. 2017 Apr 11;12(4):e0175337. doi: 10.1371/journal.pone.0175337 (PMC5388479; doi:10.1371/journal.pone.0175337)
Supplement: S1 Table — SCT = stem cell transplantation; RTE = recent thymic emigrants; TEMRA = terminally differentiated effector memory; ^ Lymphocyte counts are expressed as cells/μl (median; range). (DOC) [file pone.0175337.s002.doc]

**S1 Table**

| Immunological  variables ^ | Day +28  ATG  Yes No | | p | Day+90  ATG  Yes No | | p | Day+180  ATG  Yes No | | p |
| --- | --- | --- | --- | --- | --- | --- | --- | --- | --- |
| RTE | 0  (0-13) | 6  (0-115) | **0,0004** | 0  (0-6) | 4  (0-90) | **0,0005** | 1  (0-40) | 10  (0-65) | **0,04** |
| CD4+naïve | 1  (0-21) | 13  (0-165) | **0,0005** | 1  (0-6) | 8  (0-129) | **0,0003** | 5  (0-44) | 17  (0-69) | **0,02** |
| CD4+TEM_RA_ | 0  (0-3) | 4  (0-36) | **0,0005** | 2  (0-49) | 5  (0-41) | *0,06* | 3  (0-99) | 5  (0-59) | 0,74 |
| Treg naïve | 0  (0-2) | 1  (0-6) | **0,003** | 0  (0-1) | 1  (0-7) | **0,02** | 0  (0-3) | 0  (0-6) | 0,26 |
