# Supplementary material for: Sequential monitoring of lymphocyte subsets and of T-and-B cell neogenesis indexes to identify time-varying immunologic profiles in relation to graft-versus-host disease and relapse after allogeneic stem cell transplantation
Source: PLoS One. 2017 Apr 11;12(4):e0175337. doi: 10.1371/journal.pone.0175337 (PMC5388479; doi:10.1371/journal.pone.0175337)
Supplement: S2 Table — aGVHD = acute GVHD; SCT = stem cell transplantation; AL = acute leukaemia; MDS = myelodisplastic syndrome; CR = complete remission; upfront = never treated (all MDS); PR = partial remission; NR = no response; MUD = matched unrelated donor; MRD = matched related donor; MAC = myeloablative conditioning; RIC = reduced intensity conditioning; ATG = anti-thymocyte globulin; PB = peripheral blood; BM = bone marrow. *Patients in PR or NR at SCT were in complete remission at the first evaluation after SCT (day+30 for AL and MDS; day +60 for lymphomas) (DOC) [file pone.0175337.s003.doc]

**S2 Table**

|  | **aGVHD** | | | | |
| --- | --- | --- | --- | --- | --- |
|  | Yes (28) |  | No (22) |  |  |
| ***Characteristics*** |  | % |  | % | p |
| *Age at SCT (years)*  median (range) | 50 (17-63) |  | 48 (21-66) |  | 0.65 |
| *Sex*  male  female | 17  11 | 61  39 | 14  8 | 64  36 | 0.83 |
| *Diagnosis*  AL  MDS  Lymphomas | 14  2  12 | 50  7  43 | 14  3  5 | 64  14  22 | 0.33  0.45  0.14 |
| *Status at SCT*  CR/upfront  PR*  NR* | 15  7  6 | 54  25  21 | 15  5  2 | 68  23  9 | 0.29  0.85  0.24 |
| *Donor*  MUD  MRD | 15  13 | 54  46 | 16  6 | 73  27 | 0.17 |
| *Donor sex*  male  female | 15  13 | 54  46 | 17  5 | 77  23 | 0.08 |
| *Sex mismatch* | 15 | 54 | 9 | 41 | 0.37 |
| *Conditioning*  MAC  RIC | 11  17 | 39  61 | 9  13 | 41  59 | 0.91 |
| *ATG*  yes  no | 14  14 | 50  50 | 15  7 | 68  32 | 0.20 |
| *Source of stem cells*  PB  BM | 24  4 | 86  14 | 18  4 | 82  18 | 0.71 |
| *CD34+ cell dose (x10^6/kg)*  median (range) | 5 (1.1-5.7) |  | 5 (1.8-6.4) |  | 0.85 |
| *CD3+ cell dose (x10^7/kg)*  median (range) | 16.9 (1.2-33.3) |  | 15 (1.6-41) |  | 0.78 |
| *Bacterial infections*  *(before aGVHD)* | 6 | 21 | 8 | 36 | 0.24 |
| *CMV reactivations*  *(before aGVHD)* | 7 | 25 | 10 | 45 | 0.13 |
| *Fungal infections*  *(before aGVHD)* | 4 | 14 | 4 | 22 | 0.71 |
