# Supplementary material for: Sequential monitoring of lymphocyte subsets and of T-and-B cell neogenesis indexes to identify time-varying immunologic profiles in relation to graft-versus-host disease and relapse after allogeneic stem cell transplantation
Source: PLoS One. 2017 Apr 11;12(4):e0175337. doi: 10.1371/journal.pone.0175337 (PMC5388479; doi:10.1371/journal.pone.0175337)
Supplement: S3 Table — cGVHD = chronic GVHD; SCT = stem cell transplantation; AL = acute leukaemia; MDS = myelodisplastic syndrome; CR = complete remission; upfront = never treated (all MDS); PR = partial remission; NR = no response; MUD = matched unrelated donor; MRD = matched related donor; MAC = myeloablative conditioning; RIC = reduced intensity conditioning; ATG = anti-thymocyte globulin; PB = peripheral blood; BM = bone marrow; aGVHD = acute GVHD; ^49 evaluable patients (surviving more than 3 months) *Patients in PR or NR at SCT were in complete remission at the first evaluation after SCT (day+30 for AL and MDS; day +60 for lymphomas) (DOC) [file pone.0175337.s004.doc]

**S3 Table**

|  | **cGVHD** | | | | |
| --- | --- | --- | --- | --- | --- |
|  | Yes (13)^ |  | No (36)^ |  |  |
| ***Characteristics*** |  | % |  | % | p |
| *Age at SCT (years)*  median (range) | 50 (17-66) |  | 48 (21-63) |  | 0.82 |
| *Sex*  male  female | 9  4 | 69  31 | 21  15 | 58  42 | 0.49 |
| *Diagnosis*  AL  MDS  Lymphomas | 6  2  5 | 46  15  39 | 22  3  11 | 61  8  31 | 0.35  0.47  0.60 |
| *Status at SCT*  CR/upfront  PR*  NR* | 9  1  3 | 69  8  23 | 21  10  5 | 58  28  14 | 0.49  0.14  0.44 |
| *Donor*  MUD  MRD | 5  8 | 38  62 | 26  10 | 72  28 | **0.03** |
| *Donor sex*  male  female | 7  6 | 54  46 | 25  11 | 69  31 | 0.31 |
| *Sex mismatch* | 7 | 54 | 16 | 44 | 0.56 |
| *Conditioning*  MAC  RIC | 7  6 | 54  46 | 13  23 | 36  64 | 0.26 |
| *ATG*  yes  no | 5  8 | 38  62 | 23  13 | 64  36 | 0.11 |
| *Source of stem cells*  PB  BM | 10  3 | 77  23 | 31  5 | 86  14 | 0.44 |
| *CD34+ cell dose (x10^6/kg)*  median (range) | 4.7 (1.1-5.7) |  | 5 (1.5-6.4) |  | 0.21 |
| *CD3+ cell dose (x10^7/kg)*  median (range) | 16.2 (1.2-32.5) |  | 16.5(1.6-41) |  | 0.61 |
| *Bacterial infections*  *(before cGVHD)* | 5 | 38 | 14 | 39 | 0.98 |
| *CMV reactivations*  *(before cGVHD)* | 3 | 23 | 6 | 17 | 0.61 |
| *Fungal infections*  *(before cGVHD)* | 1 | 8 | 5 | 14 | 0.56 |
| *aGVHD*  yes  no | 11  2 | 85  15 | 16  20 | 44  56 | **0.01** |
|  |  |  |  |  |  |
