# Supplementary material for: Sequential monitoring of lymphocyte subsets and of T-and-B cell neogenesis indexes to identify time-varying immunologic profiles in relation to graft-versus-host disease and relapse after allogeneic stem cell transplantation
Source: PLoS One. 2017 Apr 11;12(4):e0175337. doi: 10.1371/journal.pone.0175337 (PMC5388479; doi:10.1371/journal.pone.0175337)
Supplement: S4 Table — SCT = stem cell transplantation; AML = acute myeloid leukaemia; ALL = acute lymphoblastic leukaemia; MDS = myelodisplastic syndrome; CR = complete remission; upfront = never treated (all MDS); PR = partial remission; NR = no response; MUD = matched unrelated donor; MRD = matched related donor; MAC = myeloablative conditioning; RIC = reduced intensity conditioning; ATG = anti-thymocyte globulin; PB = peripheral blood; BM = bone marrow; aGVHD = acute GVHD; cGVHD = chronic GVHD. *Patients in PR or NR at SCT were in complete remission at the first evaluation after SCT (day+30 for AL and MDS; day +60 for lymphomas) (DOC) [file pone.0175337.s005.doc]

**S4 Table**

|  | **Relapse** | | | | |
| --- | --- | --- | --- | --- | --- |
|  | Yes (11) |  | No (39) |  |  |
| ***Characteristics*** |  | % |  | % | p |
| *Age at SCT (years)*  median (range) | 49 (21-62) |  | 50 (17-66) |  | 0.95 |
| *Sex*  male  female | 7  4 | 64  36 | 24  15 | 61  39 | 0.90 |
| *Diagnosis*  AML  ALL  MDS  Lymphomas | 8  1  0  2 | 73  9  0  18 | 12  7  5  15 | 31  18  13  38 | **0.01**  0.48  0.21  0.20 |
| *Status at SCT*  CR/upfront  PR*  NR* | 8  1  2 | 73  9  18 | 22  11  6 | 57  28  15 | 0.33  0.19  0.82 |
| *Donor*  MUD  MRD | 9  2 | 82  18 | 22  17 | 57  43 | 0.12 |
| *Donor sex*  male  female | 8  3 | 73  27 | 24  15 | 61  39 | 0.49 |
| *Sex mismatch* | 4 | 36 | 20 | 51 | 0.38 |
| *Conditioning*  MAC  RIC | 6  5 | 54  46 | 14  25 | 36  64 | 0.26 |
| *ATG*  yes  no | 7  4 | 64  36 | 22  17 | 57  43 | 0.66 |
| *Source of stem cells*  PB  BM | 10  1 | 91  9 | 32  7 | 82  18 | 0.48 |
| *CD34+ cell dose (x10^6/kg)*  median (range) | 4.9 (1.5-5.4) |  | 5 (1.1-6.4) |  | 0.70 |
| *CD3+ cell dose (x10^7/kg)*  median (range) | 16.9 (6-27.5) |  | 16.2 (1.2-41) |  | 0.92 |
| *aGVHD*  yes  no | 4  7 | 36  64 | 24  15 | 61  39 | 0.14 |
| *cGVHD*  yes  no | 0  11 | 0  100 | 13  26 | 33  67 | 0.05 |
